
JOURNAL INFORMATION
==============================
NLM Title Abbreviation: IUCrdata
Journal ID: IUCrdata
NLM Journal ID: 101715684
Journal ID: IUCrData

IUCrData

EISSN: 2414-3146
Publisher: International Union of Crystallography

ARTICLE INFORMATION
==============================
PMCID: PMC12054753
PMID: 40337314
DOI: 10.1107/S2414314625003153
Article ID: bt4167
Article ID: S2414314625003153
Article version: 1
Subjects: Data Reports

2-(3-Hy­droxy­phen­yl)-4,5-diphenyl-1H-imidazol-3-ium hemioxalate ethanol monosolvate

C21H17N2O+·0.5C2O42−·C2H6O

https://orcid.org/0000-0001-5251-9654 Solo Peter a c *
Arockia doss M. b
a Department of Chemistry, St Joseph’s College (A) Jakhama, Kohima 797001, Nagaland, India
b Department of Chemistry, St Joseph University, Chümoukedima, Nagaland 797115, India
c https://ror.org/039543c28 Department of Environmental Studies St Xavier College, Jalukie Nagaland India
Bolte M. Editor
Goethe-Universität Frankfurt, Germany
Correspondence e-mail: solopeter82@gmail.com
Publication date: 2025 Apr
Electronic publication date: 2025 Apr 17
Volume: 10
Issue: Pt 4
Electronic Location ID: x250315
Received 2025 Apr 2; Accepted 2025 Apr 8
Copyright: © Solo and Arockia doss 2025
Copyright year: 2025
License: This is an open-access article distributed under the terms of the Creative Commons Attribution (CC-BY) Licence, which permits unrestricted use, distribution, and reproduction in any medium, provided the original authors and source are cited.
License URL: https://creativecommons.org/licenses/by/4.0/

A full version of this article is available from Crystallography Journals Online.
Page count: 3

==============================

IUCrData

ISSN 2414-3146

### 2-(3-Hydroxyphenyl)-4,5-diphenyl-1*H*-imidazol-3-ium hemioxalate ethanol monosolvate

**Peter Solo[a,c]* and M. Arockia doss[b]**

[a]Department of Chemistry, St Joseph's College (A) Jakhama, Kohima 797001, Nagaland, India, [b]Department of Chemistry, St Joseph University, Chümoukedima, Nagaland 797115, India, and [c]Department of Environmental Studies, St Xavier College, Jalukie, Nagaland, India. *Correspondence e-mail: solopeter82@gmail.com

Received 2 April 2025
Accepted 8 April 2025

Edited by M. Bolte, Goethe-Universität Frankfurt, Germany

**Keywords:** crystal structure; imidazole derivative; oxalic acid; hydrogen bonding.

**CCDC reference:** 2211???

**Structural data:** full structural data are available from iucrdata.iucr.org

In the title imidazolium oxalate solvate, $C_{21}H_{17}N_2O^+ \cdot 0.5C_2O_4^{2-} \cdot C_2H_6O$, the cation and the ethanol solvent molecule are located on general positions, whereas the complete oxalate ion is generated by a crystallographic centre of symmetry. The study confirms two proton transfers from oxalic acid to the pyrimidine-type N atoms of two separate imidazole rings. The extended structure features strong N—H···O and O—H···O hydrogen-bond interactions.

3D view

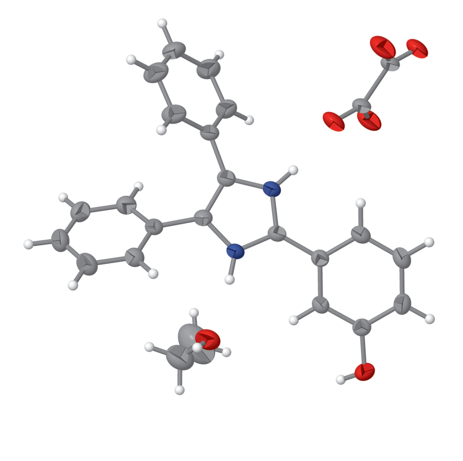

Chemical scheme

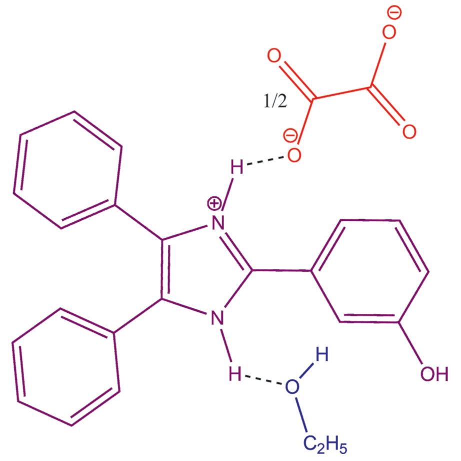

### Structure description

Compounds containing an imidazole moiety have therapeutic properties (Siwach & Verma, 2021), such as antiviral (Heinz & Vance, 1995), antihistaminic (Griffin *et al.*, 2017), antiulcer (Guerreiro *et al.*, 1990), antibacterial (Valls *et al.*, 2020), antifungal (Holt, 1976), anticancer (Kumar *et al.*, 2024), antioxidant (Pérez-González *et al.*, 2020) and antihypertension (Nikolic & Agbaba, 2012). A number of cocrystals of oxalic acids with different drugs are known (Hriňová *et al.*, 2025; Wenger & Bernstein, 2008; Othman *et al.*, 2018; Karthammaiah *et al.*, 2023), where the introduction of the cocrystallized substance improves the solubility, stability and tabletability of drugs (Chettri *et al.*, 2024).

In this article, we describe the synthesis and crystal structure of an imidazolium oxalate salt (Fig. 1). The crystal structure confirms two proton transfers from oxalic acid to the pyrimidine-type N atoms of two imidazole rings of 3-(4,5-diphenyl-1*H*-imidazol-2-yl) phenol. Each oxalate ion accepts hydrogen bonds from four protonated imidazolium cations *via* N—H···O and O—H···O interactions (Fig. 2 and Table 1). All three phenyl rings in the 2-(3-hydroxyphenyl)-4,5-diphenyl-1*H*-imidazol-3-ium cation are in different planes. The values of the torsion angles between the imidazole ring and the pendant phenyl rings are N3—C11—C6—C5 = 16.8 (2)°, N4—C17—C15—C22 = 47.4 (2)° and N3—C18—C14—C10 = 41.0 (2)°.

OPEN ACCESS
Published under a CC BY 4.0 licence

**Table 1**
Hydrogen-bond geometry (Å, °).

| $D$—H···$A$ | $D$—H | H···$A$ | $D$···$A$ | $D$—H···$A$ |
|---|---|---|---|---|
| O1—H1···O8$^{i}$ | 0.82 | 1.83 | 2.613 (3) | 161 |
| N3—H3···O7 | 0.86 | 1.87 | 2.726 (3) | 171 |
| N4—H4···O2 | 0.86 | 1.88 | 2.711 (3) | 161 |
| O7—H7···O8$^{ii}$ | 0.82 | 2.03 | 2.797 (4) | 154 |

Symmetry codes: (i) $-x+2, -y+1, -z$; (ii) $x, y-1, z$.

### Synthesis and crystallization

The synthesis of 3-(4,5-diphenyl-1*H*-imidazol-2-yl)phenol was performed by refluxing the reactants, *i.e.* benzil (0.21 g, 1 mmol), 3-hydroxybenzaldehyde (0.12 g, 1 mmol) and ammonium acetate (0.23 g, 3 mmol), in the presence of ceric ammonium nitrate (0.054 g, 0.1 mmol) as catalyst at 363 K in ethanol for about 4 h. The progress and completion of the reaction was monitored by thin-layer chromatography (TLC) using hexane–ethyl acetate solution (1:1 *v*/*v*). After the completion of the reaction, the reaction mixture was poured into ice-cold water and the precipitates were filtered off. The crude product was purified by recrystallization from a 90% ethanol solution to yield 3-(4,5-diphenyl-1*H*-imidazol-2-yl)phenol (yield: 0.23 g, 75%). Equimolar amounts of the imidazole compound and oxalic acid in an ethanol solution were heated to about 393 K and the mixture was cooled slowly to yield crystals of the title imidazolium oxalate salt.

### Refinement

Details of the crystal data, data collection and refinement are given in Table 2. H atoms were located in a difference map and refined as riding on their parent atom, with $U_{iso}$(H) = $1.5U_{eq}$(C,O) for the methyl group and the H atoms bonded to O atoms. All remaining H atoms were refined with $U_{iso}$(H) = $1.2U_{eq}$(C,N). The methyl group was allowed to rotate but not to tip. Two reflections, *i.e.* (100) and (001), were omitted as bad reflections.

**Table 2**
Experimental details.

| | |
|---|---|
| Crystal data | |
| Chemical formula | $C_{21}H_{17}N_2O^{+}\cdot0.5C_2O_4^{2-}\cdot C_2H_6O$ |
| $M_r$ | 403.44 |
| Crystal system, space group | Triclinic, $P\bar{1}$ |
| Temperature (K) | 296 |
| $a$, $b$, $c$ (Å) | 9.056 (9), 10.868 (11), 11.894 (12) |
| $\alpha$, $\beta$, $\gamma$ (°) | 77.56 (3), 72.34 (2), 69.43 (2) |
| $V$ (Å$^3$) | 1036.4 (19) |
| $Z$ | 2 |
| Radiation type | Mo $K\alpha$ |
| $\mu$ (mm$^{-1}$) | 0.09 |
| Crystal size (mm) | 0.5 × 0.5 × 0.5 |
| | |
| Data collection | |
| Diffractometer | Bruker APEXII CCD |
| Absorption correction | Multi-scan (*SADABS*; Bruker, 2016) |
| $T_{min}$, $T_{max}$ | 0.567, 0.746 |
| No. of measured, independent and observed [$I > 2\sigma(I)$] reflections | 31823, 5061, 3518 |
| $R_{int}$ | 0.058 |
| $(\sin \theta/\lambda)_{max}$ (Å$^{-1}$) | 0.695 |
| | |
| Refinement | |
| $R[F^2 > 2\sigma(F^2)]$, $wR(F^2)$, $S$ | 0.052, 0.143, 1.05 |
| No. of reflections | 5061 |
| No. of parameters | 274 |
| H-atom treatment | H-atom parameters constrained |
| $\Delta\rho_{max}$, $\Delta\rho_{min}$ (e Å$^{-3}$) | 0.37, −0.32 |

Computer programs: *APEX2* (Bruker, 2018), *SAINT* (Bruker, 2018), SHELXT (Sheldrick, 2015*a*), *SHELXL2018* (Sheldrick, 2015*b*) and *OLEX2* (Dolomanov *et al.*, 2009).

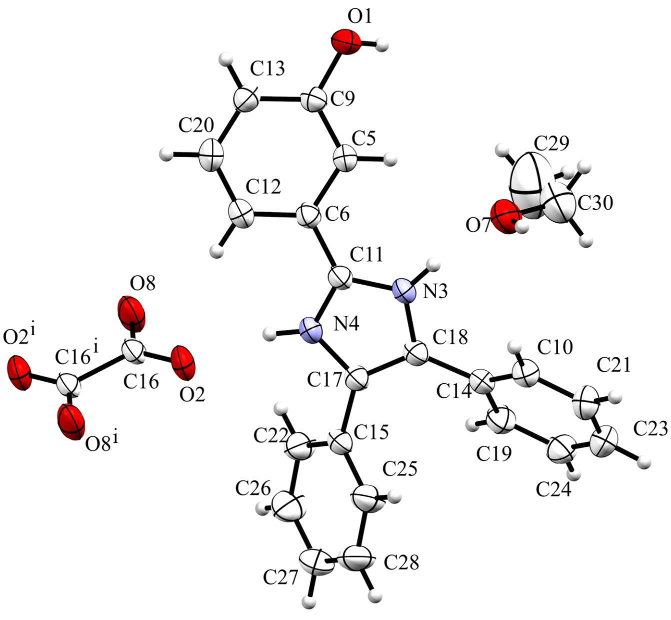

**Figure 1**
The molecular structure of the title compound, with the atomic numbering scheme. Displacement ellipsoids are drawn at the 50% probability level. [Symmetry code: (i) $-x+3, -y, -z$.]

### Acknowledgements

The authors are grateful to St Joseph's College (A) Jakhama for providing the facilities to perform the research and SAIC Tezpur University for the single-crystal XRD data collection.

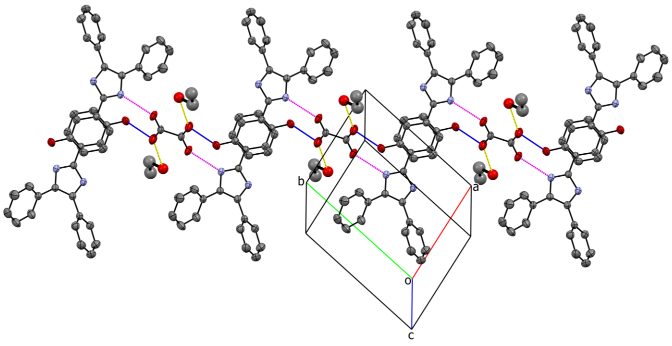

**Figure 2**
Hydrogen-bonding interactions in the title compound. N---H...O interactions are shown in magenta, O---H(imidazolium)...O interactions in blue and O---H(ethanol)...O interactions in yellow. H atoms have been omitted for clarity. Hydrogen-bonding interactions in the title compound. N—H···O interactions are in magenta and O—H···O interactions are in blue. H atoms and solvent molecules have been omitted for clarity.

### References

Bruker (2016). *SADABS*. Bruker AXS Inc., Madison, Wisconsin, USA.

Bruker (2018). *APEX2* and *SAINT*. Bruker AXS Inc., Madison, Wisconsin, USA.

Chettri, A., Subba, A., Singh, G. P. & Bag, P. P. (2024). *J. Pharm. Pharmacol.* **76**, 1–12.

Dolomanov, O. V., Bourhis, L. J., Gildea, R. J., Howard, J. A. K. & Puschmann, H. (2009). *J. Appl. Cryst.* **42**, 339–341.

Griffin, A., Hamling, K. R., Knupp, K., Hong, S., Lee, L. P. & Baraban, S. C. (2017). *Brain*, **140**, 669–683.

Guerreiro, A. S., Neves, B. C. & Quina, M. G. (1990). *Aliment. Pharmacol. Ther.* **4**, 309–313.

Heinz, B. A. & Vance, L. M. (1995). *J. Virol.* **69**, 4189–4197.

Holt, R. J. (1976). *J. Cutan. Pathol.* **3**, 45–59.

Hriňová, E., Čerňa, I., Zmeškalová, E., Ridvan, L. & Šoóš, M. (2025). *Org. Process Res. Dev.* **29**, 565–573.

Karthammaiah, G. N., Rao Amaraneni, S. & Solomon, A. K. (2023). *Acta Cryst.* E**79**, 319–322.

Kumar, A., Kaushal, A., Verma, P. K., Gupta, M. K., Chandra, G., Kumar, U., Yadav, A. K. & Kumar, D. (2024). *Eur. J. Med. Chem.* **280**, 116896.

Nikolic, K. & Agbaba, D. (2012). *Cardiovasc. Ther.* **30**, 209–216.

Othman, M. F., Anuar, N., Ad Rahman, S. & Ahmad Taifuddin, N. A. (2018). *IOP Conf. Ser. Mater. Sci. Eng.* **358**, 012065.

Pérez-González, A., García-Hernández, E. & Chigo-Anota, E. (2020). *J. Mol. Model.* **26**, 321.

Sheldrick, G. M. (2015*a*). *Acta Cryst.* A**71**, 3–8.

Sheldrick, G. M. (2015*b*). *Acta Cryst.* C**71**, 3–8.

Siwach, A. & Verma, P. K. (2021). *BMC Chem.* **15**, 12–80.

Valls, A., Andreu, J. J., Falomir, E., Luis, S. V., Atrián-Blasco, E., Mitchell, S. G. & Altava, B. (2020). *Pharmaceuticals*, **13**, 482–498.

Wenger, M. & Bernstein, J. (2008). *Cryst. Growth Des.* **8**, 1595–1598.

### full crystallographic data

*IUCrData* (2025). **10**, x250315 [https://doi.org/10.1107/S2414314625003153]

### 2-(3-Hydroxyphenyl)-4,5-diphenyl-1*H*-imidazol-3-ium hemioxalate ethanol monosolvate

Peter Solo and M. Arockia doss

### 2-(3-Hydroxyphenyl)-4,5-diphenyl-1*H*-imidazol-3-ium hemioxalate ethanol monosolvate

*Crystal data*

| | |
|---|---|
| $C_{21}H_{17}N_2O^+{\cdot}0.5C_2O_4^{2-}{\cdot}C_2H_6O$ | $Z = 2$ |
| $M_r = 403.44$ | $F(000) = 426$ |
| Triclinic, $P\overline{1}$ | $D_x = 1.293$ Mg m$^{-3}$ |
| $a = 9.056$ (9) Å | Mo $K\alpha$ radiation, $\lambda = 0.71073$ Å |
| $b = 10.868$ (11) Å | Cell parameters from 5467 reflections |
| $c = 11.894$ (12) Å | $\theta = 2.5$–$26.2°$ |
| $\alpha = 77.56$ (3)° | $\mu = 0.09$ mm$^{-1}$ |
| $\beta = 72.34$ (2)° | $T = 296$ K |
| $\gamma = 69.43$ (2)° | Block, orange |
| $V = 1036.4$ (19) Å$^3$ | 0.5 × 0.5 × 0.5 mm |

*Data collection*

| | |
|---|---|
| Bruker APEXII CCD diffractometer | 5061 independent reflections |
| $\varphi$ and $\omega$ scans | 3518 reflections with $I > 2\sigma(I)$ |
| Absorption correction: multi-scan (SADABS; Bruker, 2016) | $R_{int} = 0.058$ |
| $T_{min} = 0.957$, $T_{max} = 0.957$ | $\theta_{max} = 29.6°$, $\theta_{min} = 2.0°$ |
| 31823 measured reflections | $h = -11 \rightarrow 11$ |
| | $k = -14 \rightarrow 14$ |
| | $l = -15 \rightarrow 15$ |

*Refinement*

| | |
|---|---|
| Refinement on $F^2$ | 0 restraints |
| Least-squares matrix: full | H-atom parameters constrained |
| $R[F^2 > 2\sigma(F^2)] = 0.052$ | $w = 1/[\sigma^2(F_o^2) + (0.0644P)^2 + 0.2615P]$ where $P = (F_o^2 + 2F_c^2)/3$ |
| $wR(F^2) = 0.143$ | $(\Delta/\sigma)_{max} < 0.001$ |
| $S = 1.05$ | $\Delta\rho_{max} = 0.37$ e Å$^{-3}$ |
| 5061 reflections | $\Delta\rho_{min} = -0.32$ e Å$^{-3}$ |
| 274 parameters | |

*Special details*

**Geometry**. All esds (except the esd in the dihedral angle between two l.s. planes) are estimated using the full covariance matrix. The cell esds are taken into account individually in the estimation of esds in distances, angles and torsion angles; correlations between esds in cell parameters are only used when they are defined by crystal symmetry. An approximate (isotropic) treatment of cell esds is used for estimating esds involving l.s. planes.

*Fractional atomic coordinates and isotropic or equivalent isotropic displacement parameters (Å$^2$)*

| | *x* | *y* | *z* | $U_{iso}$*/$U_{eq}$ |
|---|---|---|---|---|
| O1 | 1.23020 (14) | 0.27909 (12) | −0.20771 (11) | 0.0444 (3) |
| H1 | 1.238649 | 0.221040 | −0.151031 | 0.067* |
| O2 | 0.50008 (16) | 0.83543 (11) | 0.03285 (11) | 0.0442 (3) |
| N3 | 0.66676 (16) | 0.36104 (12) | 0.11752 (12) | 0.0314 (3) |
| H3 | 0.720090 | 0.282619 | 0.098274 | 0.038* |
| N4 | 0.58706 (16) | 0.57339 (12) | 0.11283 (12) | 0.0312 (3) |
| H4 | 0.580136 | 0.655501 | 0.090389 | 0.037* |
| C5 | 0.96646 (19) | 0.37050 (15) | −0.07708 (14) | 0.0312 (3) |
| H5 | 0.970348 | 0.288935 | −0.031343 | 0.037* |
| C6 | 0.83192 (19) | 0.48199 (15) | −0.04782 (14) | 0.0291 (3) |
| O7 | 0.82059 (18) | 0.10267 (13) | 0.08045 (14) | 0.0559 (4) |
| H7 | 0.772174 | 0.068017 | 0.054917 | 0.084* |
| O8 | 0.67298 (17) | 0.92601 (12) | 0.05650 (14) | 0.0534 (4) |
| C9 | 1.09467 (19) | 0.38249 (16) | −0.17525 (14) | 0.0318 (4) |
| C10 | 0.4602 (2) | 0.18315 (16) | 0.26039 (16) | 0.0368 (4) |
| H10 | 0.496326 | 0.173701 | 0.179802 | 0.044* |
| C11 | 0.69811 (18) | 0.47219 (15) | 0.05714 (14) | 0.0293 (3) |
| C12 | 0.8266 (2) | 0.60359 (16) | −0.11698 (15) | 0.0367 (4) |
| H12 | 0.738378 | 0.678219 | −0.096977 | 0.044* |
| C13 | 1.0869 (2) | 0.50419 (17) | −0.24494 (15) | 0.0385 (4) |
| H13 | 1.171617 | 0.511972 | −0.311018 | 0.046* |
| C14 | 0.47346 (19) | 0.28902 (15) | 0.30020 (14) | 0.0318 (4) |
| C15 | 0.35217 (19) | 0.61249 (15) | 0.29465 (15) | 0.0322 (4) |
| C16 | 0.5498 (2) | 0.93044 (15) | 0.02620 (15) | 0.0336 (4) |
| C17 | 0.48478 (19) | 0.52616 (15) | 0.21215 (14) | 0.0310 (3) |
| C18 | 0.53606 (19) | 0.39090 (15) | 0.21533 (14) | 0.0312 (3) |
| C19 | 0.4229 (2) | 0.29914 (17) | 0.42203 (15) | 0.0401 (4) |
| H19 | 0.433637 | 0.368164 | 0.450264 | 0.048* |
| C20 | 0.9534 (2) | 0.61304 (17) | −0.21584 (16) | 0.0416 (4) |
| H20 | 0.948217 | 0.693904 | −0.263119 | 0.050* |
| C21 | 0.3938 (2) | 0.09172 (18) | 0.33962 (18) | 0.0469 (5) |
| H21 | 0.384967 | 0.021362 | 0.312113 | 0.056* |
| C22 | 0.3787 (2) | 0.70962 (18) | 0.33806 (18) | 0.0469 (5) |
| H22 | 0.478999 | 0.725234 | 0.310859 | 0.056* |
| C23 | 0.3404 (2) | 0.10459 (19) | 0.45989 (18) | 0.0497 (5) |
| H23 | 0.293561 | 0.044057 | 0.512833 | 0.060* |
| C24 | 0.3567 (2) | 0.20705 (19) | 0.50099 (17) | 0.0469 (5) |
| H24 | 0.323298 | 0.214432 | 0.581960 | 0.056* |
| C25 | 0.2006 (2) | 0.59230 (19) | 0.33520 (18) | 0.0453 (5) |
| H25 | 0.181223 | 0.528125 | 0.305996 | 0.054* |
| C26 | 0.2560 (3) | 0.7831 (2) | 0.4217 (2) | 0.0572 (6) |
| H26 | 0.274879 | 0.847358 | 0.451212 | 0.069* |
| C27 | 0.1063 (2) | 0.7623 (2) | 0.46189 (19) | 0.0529 (5) |
| H27 | 0.024275 | 0.812452 | 0.517990 | 0.064* |
| C28 | 0.0785 (2) | 0.6664 (2) | 0.4183 (2) | 0.0552 (5) |

| | | | | |
|---|---|---|---|---|
| H28 | −0.022498 | 0.651913 | 0.445019 | 0.066* |
| C29 | 0.9744 (6) | 0.0693 (4) | 0.2205 (4) | 0.1308 (15) |
| H29A | 1.027149 | 0.004373 | 0.276394 | 0.196* |
| H29B | 0.898109 | 0.143431 | 0.259980 | 0.196* |
| H29C | 1.054539 | 0.098600 | 0.157279 | 0.196* |
| C30 | 0.8899 (3) | 0.0118 (2) | 0.1725 (3) | 0.0749 (7) |
| H30A | 0.803803 | −0.011942 | 0.235633 | 0.090* |
| H30B | 0.964940 | −0.068401 | 0.140023 | 0.090* |

*Atomic displacement parameters (Å$^2$)*

| | $U^{11}$ | $U^{22}$ | $U^{33}$ | $U^{12}$ | $U^{13}$ | $U^{23}$ |
|---|---|---|---|---|---|---|
| O1 | 0.0326 (6) | 0.0396 (7) | 0.0446 (7) | −0.0014 (5) | 0.0002 (5) | −0.0021 (5) |
| O2 | 0.0544 (8) | 0.0217 (6) | 0.0555 (8) | −0.0086 (5) | −0.0191 (6) | 0.0001 (5) |
| N3 | 0.0321 (7) | 0.0215 (6) | 0.0356 (7) | −0.0052 (5) | −0.0039 (6) | −0.0050 (5) |
| N4 | 0.0310 (7) | 0.0219 (6) | 0.0374 (7) | −0.0060 (5) | −0.0057 (6) | −0.0049 (5) |
| C5 | 0.0322 (8) | 0.0249 (7) | 0.0347 (8) | −0.0086 (6) | −0.0080 (7) | −0.0008 (6) |
| C6 | 0.0299 (8) | 0.0261 (7) | 0.0317 (8) | −0.0084 (6) | −0.0080 (7) | −0.0044 (6) |
| O7 | 0.0552 (9) | 0.0370 (7) | 0.0726 (10) | −0.0130 (6) | −0.0076 (7) | −0.0147 (7) |
| O8 | 0.0493 (8) | 0.0311 (7) | 0.0812 (10) | −0.0078 (6) | −0.0318 (8) | 0.0048 (6) |
| C9 | 0.0281 (8) | 0.0309 (8) | 0.0349 (8) | −0.0072 (6) | −0.0078 (7) | −0.0044 (7) |
| C10 | 0.0377 (9) | 0.0317 (8) | 0.0386 (9) | −0.0101 (7) | −0.0071 (7) | −0.0038 (7) |
| C11 | 0.0288 (8) | 0.0243 (7) | 0.0332 (8) | −0.0053 (6) | −0.0081 (7) | −0.0049 (6) |
| C12 | 0.0377 (9) | 0.0260 (8) | 0.0416 (9) | −0.0054 (7) | −0.0099 (8) | −0.0015 (7) |
| C13 | 0.0354 (9) | 0.0407 (9) | 0.0368 (9) | −0.0155 (8) | −0.0049 (7) | 0.0013 (7) |
| C14 | 0.0270 (8) | 0.0264 (8) | 0.0368 (9) | −0.0045 (6) | −0.0055 (7) | −0.0026 (6) |
| C15 | 0.0293 (8) | 0.0263 (8) | 0.0375 (9) | −0.0044 (6) | −0.0065 (7) | −0.0065 (7) |
| C16 | 0.0356 (9) | 0.0218 (8) | 0.0359 (9) | −0.0021 (7) | −0.0056 (7) | −0.0050 (6) |
| C17 | 0.0290 (8) | 0.0271 (8) | 0.0356 (8) | −0.0068 (6) | −0.0075 (7) | −0.0052 (6) |
| C18 | 0.0290 (8) | 0.0274 (8) | 0.0347 (8) | −0.0059 (6) | −0.0059 (7) | −0.0064 (6) |
| C19 | 0.0415 (10) | 0.0358 (9) | 0.0390 (9) | −0.0085 (8) | −0.0068 (8) | −0.0071 (7) |
| C20 | 0.0477 (10) | 0.0310 (9) | 0.0427 (10) | −0.0140 (8) | −0.0108 (8) | 0.0048 (7) |
| C21 | 0.0505 (11) | 0.0352 (9) | 0.0558 (12) | −0.0181 (8) | −0.0111 (9) | −0.0023 (8) |
| C22 | 0.0411 (10) | 0.0417 (10) | 0.0575 (11) | −0.0182 (8) | 0.0044 (9) | −0.0198 (9) |
| C23 | 0.0457 (11) | 0.0422 (10) | 0.0516 (12) | −0.0170 (9) | −0.0028 (9) | 0.0061 (9) |
| C24 | 0.0450 (11) | 0.0468 (11) | 0.0366 (9) | −0.0084 (9) | −0.0022 (8) | −0.0012 (8) |
| C25 | 0.0318 (9) | 0.0438 (10) | 0.0610 (12) | −0.0082 (8) | −0.0083 (9) | −0.0184 (9) |
| C26 | 0.0585 (13) | 0.0478 (11) | 0.0643 (13) | −0.0207 (10) | 0.0064 (11) | −0.0287 (10) |
| C27 | 0.0445 (11) | 0.0450 (11) | 0.0540 (12) | −0.0036 (9) | 0.0056 (9) | −0.0181 (9) |
| C28 | 0.0296 (9) | 0.0576 (12) | 0.0709 (14) | −0.0099 (9) | 0.0020 (9) | −0.0192 (11) |
| C29 | 0.171 (4) | 0.104 (3) | 0.156 (4) | −0.061 (3) | −0.094 (3) | 0.012 (2) |
| C30 | 0.0766 (17) | 0.0528 (14) | 0.0881 (19) | −0.0125 (12) | −0.0223 (15) | −0.0038 (13) |

*Geometric parameters (Å, º)*

| | | | |
|---|---|---|---|
| O1—C9 | 1.359 (2) | C15—C25 | 1.389 (3) |
| O1—H1 | 0.8200 | C15—C17 | 1.475 (2) |
| O2—C16 | 1.243 (2) | C16—C16$^{i}$ | 1.570 (3) |

| | | | |
|---|---|---|---|
| N3—C11 | 1.341 (2) | C17—C18 | 1.373 (3) |
| N3—C18 | 1.385 (2) | C19—C24 | 1.385 (3) |
| N3—H3 | 0.8600 | C19—H19 | 0.9300 |
| N4—C11 | 1.339 (2) | C20—H20 | 0.9300 |
| N4—C17 | 1.388 (2) | C21—C23 | 1.385 (3) |
| N4—H4 | 0.8600 | C21—H21 | 0.9300 |
| C5—C9 | 1.395 (2) | C22—C26 | 1.382 (3) |
| C5—C6 | 1.400 (2) | C22—H22 | 0.9300 |
| C5—H5 | 0.9300 | C23—C24 | 1.376 (3) |
| C6—C12 | 1.392 (2) | C23—H23 | 0.9300 |
| C6—C11 | 1.468 (2) | C24—H24 | 0.9300 |
| O7—C30 | 1.443 (3) | C25—C28 | 1.379 (3) |
| O7—H7 | 0.8200 | C25—H25 | 0.9300 |
| O8—C16 | 1.255 (2) | C26—C27 | 1.376 (3) |
| C9—C13 | 1.392 (3) | C26—H26 | 0.9300 |
| C10—C21 | 1.382 (3) | C27—C28 | 1.384 (3) |
| C10—C14 | 1.389 (3) | C27—H27 | 0.9300 |
| C10—H10 | 0.9300 | C28—H28 | 0.9300 |
| C12—C20 | 1.387 (3) | C29—C30 | 1.434 (4) |
| C12—H12 | 0.9300 | C29—H29A | 0.9600 |
| C13—C20 | 1.378 (3) | C29—H29B | 0.9600 |
| C13—H13 | 0.9300 | C29—H29C | 0.9600 |
| C14—C19 | 1.397 (3) | C30—H30A | 0.9700 |
| C14—C18 | 1.476 (2) | C30—H30B | 0.9700 |
| C15—C22 | 1.388 (3) | | |

| | | | |
|---|---|---|---|
| C9—O1—H1 | 109.5 | N3—C18—C14 | 122.98 (14) |
| C11—N3—C18 | 110.12 (13) | C24—C19—C14 | 120.41 (17) |
| C11—N3—H3 | 124.9 | C24—C19—H19 | 119.8 |
| C18—N3—H3 | 124.9 | C14—C19—H19 | 119.8 |
| C11—N4—C17 | 109.91 (14) | C13—C20—C12 | 120.86 (17) |
| C11—N4—H4 | 125.0 | C13—C20—H20 | 119.6 |
| C17—N4—H4 | 125.0 | C12—C20—H20 | 119.6 |
| C9—C5—C6 | 119.62 (15) | C10—C21—C23 | 120.18 (18) |
| C9—C5—H5 | 120.2 | C10—C21—H21 | 119.9 |
| C6—C5—H5 | 120.2 | C23—C21—H21 | 119.9 |
| C12—C6—C5 | 119.88 (15) | C26—C22—C15 | 120.00 (18) |
| C12—C6—C11 | 119.70 (14) | C26—C22—H22 | 120.0 |
| C5—C6—C11 | 120.40 (15) | C15—C22—H22 | 120.0 |
| C30—O7—H7 | 109.5 | C24—C23—C21 | 119.94 (18) |
| O1—C9—C13 | 117.16 (15) | C24—C23—H23 | 120.0 |
| O1—C9—C5 | 122.82 (15) | C21—C23—H23 | 120.0 |
| C13—C9—C5 | 120.01 (15) | C23—C24—C19 | 120.14 (19) |
| C21—C10—C14 | 120.50 (18) | C23—C24—H24 | 119.9 |
| C21—C10—H10 | 119.7 | C19—C24—H24 | 119.9 |
| C14—C10—H10 | 119.7 | C28—C25—C15 | 120.71 (18) |
| N4—C11—N3 | 107.12 (15) | C28—C25—H25 | 119.6 |
| N4—C11—C6 | 125.96 (15) | C15—C25—H25 | 119.6 |

| | | | |
|---|---|---|---|
| N3—C11—C6 | 126.88 (14) | C27—C26—C22 | 120.74 (19) |
| C20—C12—C6 | 119.72 (16) | C27—C26—H26 | 119.6 |
| C20—C12—H12 | 120.1 | C22—C26—H26 | 119.6 |
| C6—C12—H12 | 120.1 | C26—C27—C28 | 119.60 (18) |
| C20—C13—C9 | 119.88 (16) | C26—C27—H27 | 120.2 |
| C20—C13—H13 | 120.1 | C28—C27—H27 | 120.2 |
| C9—C13—H13 | 120.1 | C25—C28—C27 | 119.95 (19) |
| C10—C14—C19 | 118.78 (15) | C25—C28—H28 | 120.0 |
| C10—C14—C18 | 120.73 (16) | C27—C28—H28 | 120.0 |
| C19—C14—C18 | 120.47 (15) | C30—C29—H29A | 109.5 |
| C22—C15—C25 | 118.99 (16) | C30—C29—H29B | 109.5 |
| C22—C15—C17 | 120.96 (16) | H29A—C29—H29B | 109.5 |
| C25—C15—C17 | 119.95 (15) | C30—C29—H29C | 109.5 |
| O2—C16—O8 | 126.35 (16) | H29A—C29—H29C | 109.5 |
| O2—C16—C16$^{i}$ | 117.1 (2) | H29B—C29—H29C | 109.5 |
| O8—C16—C16$^{i}$ | 116.57 (18) | C29—C30—O7 | 111.2 (2) |
| C18—C17—N4 | 106.51 (14) | C29—C30—H30A | 109.4 |
| C18—C17—C15 | 130.12 (15) | O7—C30—H30A | 109.4 |
| N4—C17—C15 | 123.35 (15) | C29—C30—H30B | 109.4 |
| C17—C18—N3 | 106.31 (14) | O7—C30—H30B | 109.4 |
| C17—C18—C14 | 130.72 (15) | H30A—C30—H30B | 108.0 |

| | | | |
|---|---|---|---|
| C9—C5—C6—C12 | 0.3 (2) | C15—C17—C18—N3 | −179.02 (16) |
| C9—C5—C6—C11 | −178.19 (14) | N4—C17—C18—C14 | −179.89 (16) |
| C6—C5—C9—O1 | 178.87 (15) | C15—C17—C18—C14 | 1.5 (3) |
| C6—C5—C9—C13 | −1.3 (2) | C11—N3—C18—C17 | 1.21 (18) |
| C17—N4—C11—N3 | 1.29 (18) | C11—N3—C18—C14 | −179.25 (15) |
| C17—N4—C11—C6 | −176.79 (15) | C10—C14—C18—C17 | 138.40 (19) |
| C18—N3—C11—N4 | −1.55 (18) | C19—C14—C18—C17 | −40.1 (3) |
| C18—N3—C11—C6 | 176.51 (15) | C10—C14—C18—N3 | −41.0 (2) |
| C12—C6—C11—N4 | −17.6 (2) | C19—C14—C18—N3 | 140.47 (17) |
| C5—C6—C11—N4 | 160.91 (16) | C10—C14—C19—C24 | −1.7 (3) |
| C12—C6—C11—N3 | 164.72 (16) | C18—C14—C19—C24 | 176.82 (16) |
| C5—C6—C11—N3 | −16.8 (2) | C9—C13—C20—C12 | 0.6 (3) |
| C5—C6—C12—C20 | 1.2 (3) | C6—C12—C20—C13 | −1.7 (3) |
| C11—C6—C12—C20 | 179.69 (16) | C14—C10—C21—C23 | −0.3 (3) |
| O1—C9—C13—C20 | −179.32 (16) | C25—C15—C22—C26 | 1.0 (3) |
| C5—C9—C13—C20 | 0.9 (3) | C17—C15—C22—C26 | −175.42 (19) |
| C21—C10—C14—C19 | 1.8 (3) | C10—C21—C23—C24 | −1.4 (3) |
| C21—C10—C14—C18 | −176.68 (16) | C21—C23—C24—C19 | 1.6 (3) |
| C11—N4—C17—C18 | −0.55 (18) | C14—C19—C24—C23 | 0.0 (3) |
| C11—N4—C17—C15 | 178.19 (15) | C22—C15—C25—C28 | −0.7 (3) |
| C22—C15—C17—C18 | 131.0 (2) | C17—C15—C25—C28 | 175.74 (18) |
| C25—C15—C17—C18 | −45.4 (3) | C15—C22—C26—C27 | −0.8 (3) |
| C22—C15—C17—N4 | −47.4 (2) | C22—C26—C27—C28 | 0.2 (4) |

| | | | |
|---|---|---|---|
| C25—C15—C17—N4 | 136.21 (18) | C15—C25—C28—C27 | 0.2 (3) |
| N4—C17—C18—N3 | −0.39 (17) | C26—C27—C28—C25 | 0.1 (3) |

Symmetry code: (i) $-x+1, -y+2, -z$.

*Hydrogen-bond geometry (Å, º)*

| *D*—H···*A* | *D*—H | H···*A* | *D*···*A* | *D*—H···*A* |
|---|---|---|---|---|
| O1—H1···O8$^{ii}$ | 0.82 | 1.83 | 2.613 (3) | 161 |
| N3—H3···O7 | 0.86 | 1.87 | 2.726 (3) | 171 |
| N4—H4···O2 | 0.86 | 1.88 | 2.711 (3) | 161 |
| O7—H7···O8$^{iii}$ | 0.82 | 2.03 | 2.797 (4) | 154 |

Symmetry codes: (ii) $-x+2, -y+1, -z$; (iii) $x, y-1, z$.
